# Graphene-cobalt hexacyanoferrate modified sensor doped with molecularly imprinted polymer for selective potentiometric determination of bupropion

**DOI:** 10.1038/s41598-025-16259-z

**Published:** 2025-08-22

**Authors:** Eman M. Moaaz, Ahmed S. Fayed, Mamdouh R. Rezk, Ezzat M. Abdel-Moety

**Affiliations:** Pharmaceutical Analytical Chemistry Department, Faculty of Pharmacy, Cairo University, Kasr El-Aini Street, Cairo, ET-11562 Egypt

**Keywords:** Bupropion, Cobalt hexacyanoferrate, Graphene, Molecularly imprinted polymer, Solid-Contact ion selective electrode, Sensors, Graphene, Nanoparticles, Characterization and analytical techniques

## Abstract

This study presents the first application of a graphene/cobalt hexacyanoferrate composite as an ion-to-electron transducer interlayer for the selective electrochemical determination of bupropion. The composite was prepared by homogeneously dispersing graphene with tween 80 and then decorating it with cobalt-hexacyanoferrate nanoparticles. Modifying glassy carbon electrodes with this interlayer improved and stabilized the measured potential by preventing the formation of an aqueous layer beneath the sensing membrane and enhancing charge transfer. The formation of the aqueous layer is a phenomenon commonly associated with solid-contact ion-selective electrodes. These electrodes encounter challenges regarding selectivity affecting their analytical performance in the presence of ions with similar charges and proper lipophilicity. Molecularly imprinted polymer (MIP) approach was employed to resolve the selectivity problem of analyzing bupropion in presence of naltrexone for obesity management. Scanning electron microscope and Fourier-transform infrared were utilized to characterize the fabricated composite and the MIP. Two cationic exchangers were separately integrated with the precipitated MIP to produce sensors for selective determination of bupropion in the dosage form and in spiked human plasma. The sensors exhibited Nernstian slope of 54.66 mV/decade and 55.89 mV/decade with detection limits as low as 2.51 × 10^− 7^ M and 2.0 × 10^− 7^ M. Moreover, three different metric ways verified sufficient sustainability of the proposed potentiometric method.

**Supplementary Information:**

The online version contains supplementary material available at 10.1038/s41598-025-16259-z.

## Introduction

The simplicity of electrochemical analysis methods offers an alternative approach to conventional methods in analyte analysis. Ion-selective electrodes (ISEs) are commonly used due to their low cost, portability, rapidity, and permission to directly assay analyte ion activity without prior separation or sample preparation^1^. Moreover, the instrumentation required for ISEs is neither sophisticated nor costly, enhancing their practicality over chromatographic techniques^2–9^. Solid contact ion-selective electrodes (SC-ISEs) are promising surrogates to conventional liquid contact ion-selective electrodes for their easy handling and maintenance^10–13^. However, SC-ISEs suffer from irregular potential drift and standard potential (*E*^*o*^) irreproducibility, probably due to the aqueous layer formation between the ion-selective membrane (ISM) and the electrode surface, limiting their use. That aqueous layer acts as a reservoir for electrolytes upon altering the composition of the tested samples, affecting the suitable interface between the sensing films^14–16^. To overcome those limitations, various transducers have been introduced between the ISM and the solid conductor, such as conducting polymers^17^carbon nanotubes (CNTs)^18^and graphene (GR) and reduced graphene oxide^19–21^.

Graphene (GR) is a very hydrophobic material present as a layer or few layers of graphite in a honeycomb-like structure with outstanding electronic and electrochemical properties and high chemical stability^22,23^. Those characteristics made GR a promising material for designing electrochemical sensors and biosensors^20,23^. Various methods were developed for GR- dispersal; oxidation to graphene oxide was introduced with Hummers’ method^24^. Aqueous graphene dispersion can be prepared using non-ionic surfactants above the critical micelle concentration to decrease the surface tension between water and GR-particles. Guardia et al.^25^ used different surfactants in GR-dispersion in 0.5% and 1% concentrations. The results revealed that some non-ionic surfactants developed more stable homogenous GR-dispersion. GR was used as a transducer layer in ion-selective sensors and biosensors to detect many pharmaceutical, biological, and environmental analytes^26–29^ and cancer markers with metal cyanide derivative nanoparticles^30^.

Metal hexacyanoferrates ([Fe(CN)_6_]^n−^), have captured attention for their magnetic and electrochemical properties. They were introduced to electrochemistry, starting with Prussian blue particles as electron transfer mediators^31,32^. Following the investigation of cobalt hexacyanoferrate (CoHCF) through electrochemical and spectrochemical characterizations^33^there was a particular interest in fabricating various electrodes, such as H_2_O_2_ and glucose biosensors, using CoHCF-nanoparticles (CoNP)^34^. The improvement of the electron transfer process by combining CoNP with CNTs encouraged the investigation of the synergistic effect between CoNP and GR in the electrochemical detection of prostate-specific antigen, resulting in a highly sensitive sensor with a low detection limit^30^. Moreover, CoHCF/GR heterostructure was used in the amperometric determination of captopril^27^.

The effectiveness of the ISM relies on an ion exchanger’s function, which allows the counter ion to be replaced by the target analyte in its ionic form. Ion exchangers are typically categorized as cationic for positively charged analytes or anionic for negatively charged ones. However, the presence of other ions in the sample with the same charge and an optimal level of lipophilicity can interfere with the accurate detection of the target analyte. To address this issue, selective carriers like ionophores and custom-designed molecularly imprinted polymers (MIPs) are incorporated to enhance selectivity^35^.

MIPs are specialized polymers produced through interactions between functional monomers with acidic or basic groups and a target template, typically using a crosslinking monomer and an initiator^36^. Following the non-covalent copolymerization process, the template molecules are removed, leaving recognition cavities that mirror the template’s shape, size, and functional group arrangement. The high cross-linker content preserves the structural memory of these cavities, ensuring the functional groups remain positioned to effectively recognize and bind to the target molecules^37^. The interaction between these imprinted sites and target analytes is similar to antigen-antibody binding, involving hydrogen bonds, hydrophobic forces, ionic interactions, and Van der Waals forces. MIPs are known as synthetic receptors that offer advantages such as affordability, direct synthesis with multiple methods, chemical and thermal stability, ease of handling, and reusability without significant activity loss^38^. These benefits make MIPs valuable in a range of applications, including separation techniques^39^electrochemical methods^40–44^and analyses in biological and food samples^36,45^.

Bupropion (BUP) is an antidepressant drug that is also used for smoking cessation. It exerts its action through inhibiting the reuptake of norepinephrine and dopamine neurotransmitters^46^. It is combined with naltrexone (NAL), an antagonist for opioid receptors^47^for obesity management *via* regulating energy balance and reducing food craving^48^. Numerous methods were reported for the determination of the combined drugs, including chromatographic^49–54^ and UV- spectrophotometric methods^55,56^. Few electrochemical methods were developed for BUP analysis as a single analyte: one of them did not resolve BUP in presence of other interfering drugs^57^ and four voltammetric methods^58–61^.

This work presents a new sensor for the analysis of BUP in the presence of NAL in a pharmaceutical formulation (Contrave^®^ tablets) for obesity management, as well as in spiked human plasma. Selective determination of BUP was achieved with the utilization of MIP. Initially, the selection of the cationic exchanger was inspected for both drugs. Two glassy carbon electrodes (GCE), with different cationic exchangers, modified with graphene/cobalt hexacyanoferrate composite (GCC), were used for BUP-selective analysis with the integration of the fabricated BUP-MIP. To the best of our knowledge, the GCC has been prepared, characterized, and used for the first time, as an ion-to-electron transducer layer to improve both potential steadiness and standard potential reproducibility. The selectivity of the sensors was estimated using NAL in comparison with MIP-free sensors, in addition to commonly used cations and additives. Three assessment approaches evaluated the sustainability of the method: Analytical GREEnness metric tool (AGREE)^62^White Analytical Chemistry (WAC) approach^63^and Modified Green Analytical Procedure Index (MoGAPI)^64^.

## Experimental

### Materials and methods

#### Chemicals and reagents

BUP (BN: ACBUPNF016) was kindly provided by Sun Pharmaceutical Industries Ltd (Giza, Egypt). NAL (BN: PDNRHNF002) was gently provided by Eva Pharma (Giza, Egypt). BUP potency was checked by a reported high-performance liquid chromatography (HPLC) method^51^ and was found to be 100.08 ± 1.98%. Contrave^®^ tablets (BN: E1687A) (Orexigen Therapeutics Inc., California, USA) were obtained from Canadian local market. Each tablet claimed to contain 90 mg BUP combined with 8 mg NAL. All the chemicals used were of high analytical grade. Azobisisobutyronitrile (AIBN), calix[6]arene (CX6), dimethylsulfoxide (DMSO), ethylene glycol dimthacrylate (EGDMA), ethanol, methanol, glacial acetic acid, methacrylic acid (MAA), polyvinyl chloride (PVC), potassium tetrakis (*p*-chlorophenyl) borate (K-TCPB), potassium tetrakis [3,5-bis(trifluoromethyl)phenyl] borate (K-TFMPB), sodium tetraphenylborate (TPB), phosphomolybdic acid (PMA), 2-nitrophenyl octyl ether (NPOE), tetrahydrofuran (THF), KCl, NaCl, potassium ferrocyanide & ferricyanide (K_4_[Fe(CN)_6_] & K_3_[Fe(CN)_6_]) were obtained from Sigma Aldrich (MO, USA). Di-sodium hydrogen phosphate was obtained from E. Merck (Darmstadt, Germany). Ortho-phosphoric acid was from SD Fine-Chem. Ltd Company (Mumbai, India). Graphene nano-platelets (GR) (6–8 nm thick × 5 μm wide) were purchased from Strem Chemicals INC. (Newburyport, USA). Tween 80, CoCl_2_, CaCl_2_, MgCl_2_ were purchased from El-Nasr Pharmaceutical Chemicals Co. (Cairo, Egypt). Ultra-pure HPLC grade water purified by New Human Power 1 device, Human Corporation (Seoul, Korea). Human plasma was purchased from the Holding Company for Biological Products and Vaccines (VACSERA) (Cairo, Egypt).

#### Instruments

A Jenway digital ion analyzer model 3540 (Essex, UK) coupled with Ag/AgCl double-junction reference electrode (Thermo Scientific Orion 900200, MA, USA); 10% KNO_3_ is used as a bridge electrolyte and 3 M KCl is the inner filling solution) to measure the potential difference. A Jenway pH glass electrode (Essex, UK) was used for pH-adjustments. Glassy carbon electrodes (GCE) (OD: 10 mm, ID: 5 mm) as working electrodes (CH Instruments, Austin, USA). Sigma centrifuge device (Focus Scientific, Ireland). Field-emission scanning electron microscope (SEM) FEG model Quanta 250, Fei Company (Oregon, USA) was used for MIP and NIP surface imaging. Sigma 500 VP SEM (Carl Zeiss, Germany) was used for the composite surface imaging. Brunauere-Emmette-Teller (BET) analyzer model BELSORP (Microtrac, Osaka, Japan). IR-spectrometer model 1310 (Perkin- Elmer, Norwalk, USA). Metrohm Autolab potentiostat PGSTAT204 (Utrecht, Netherland) operated with Nova 1.11 software has been used to perform electrochemical impedance measurements. The dynamic size of the composite was measured using a Zetasizer (Malvern INS., UK) via dynamic light scattering technique (DLS).

### Synthesis of MIP for BUP

The MIP was synthesized using the precipitation-polymerization method^42,65^. In a 50-mL glass-capped bottle, 0.5 mmol of BUP serving as a template was dissolved in 40 mL of DMSO as a porogenic solvent. Following this, 2 mmol of the functional monomer MAA was added, and the solution was sonicated for 15 min. Nextly, 8 mmol of EGDMA cross-linker and 0.6 mmol of initiator AIBN were added and sonicated for 1 min. The solution was then purged with nitrogen gas for 15 min and placed in a thermostatic oil bath at 60 °C for 24 h. The MIP precipitate was filtered by decantation to remove excess solvent, followed by repeated washing with ethanol and centrifugation to eliminate unreacted materials. To extract the template, the polymer was washed several times with a methanol and acetic acid mixture (9: 1, v/v). The successful removal of BUP was confirmed through UV/Vis-spectroscopy, as no drug peaks (typically around 250 nm) were detected in the last effluent [Fig. S1]. Under acetic acid conditions similar to previously reported^54^BUP exhibits a major peak at 250 nm, unlike in methanol where the peak at 210 nm was the uppermost one. No peaks were detected in the last effluent at 250 nm, supporting the complete template removal. Finally, the MIP was rinsed with distilled water until a neutral pH was achieved, then dried at 100 °C for 2 h. The non-imprinted polymer (NIP) was prepared similarly, excluding the template addition.

### Brunauer-Emmett-Teller (BET) surface area and porosity analysis

The polymers were subjected to initial cleaning and degassing to get rid of adsorbed moisture prior to analysis by nitrogen gas flow at 100 °C for 4 h. The adsorption/desorption isotherms were then measured at -196 °C by flow of liquid nitrogen.

### Preparations for binding capacity calculation

The functionality of the leached MIP as a specific recognition ionophore is determined by its competency to rebind with its template. A UV spectrophotometric method of BUP and NAL^66^ was performed to assess the binding capacity of the polymers towards the studied and co-formulated interfering drugs. The binding capacity was assessed by adding 5.0 mg of the MIP or NIP into a 10-mL volumetric flask containing 0.027 mM of BUP dissolved in phosphate buffer pH 5. The mixtures were stirred for 2 h to allow incubation, followed by centrifugation at 4000 rpm for 15 min. The clear supernatants were then passed through a 0.22 μm syringe filter and injected into the HPLC column for analysis.

### Preparation of graphene-cobalt hexacyanoferrate composite (GCC)

Aqueous GR dispersions were prepared following a previously reported method^25^ using a non-ionic surfactant tween 80. GR powder (200 mg) was sonicated in 5 mL of a 1% w/v aqueous solutions tween 80 for 2 h to achieve homogenous dispersion. The dispersion was centrifuged at 4000 rpm for 15 min. to sediment the thick particles and collect the supernatant containing the dispersed graphene nanoparticles. The GCC nanoparticles were synthesized based on Yang et al.. procedure with some modifications^34^ and stepped as reported^27^combining the results of the three mentioned methods into a unified approach. Briefly, a solution of 40.8 mg K_3_[Fe(CN)_6_] and 9.3 mg KCl in 10.0 mL water was added to the GR dispersion with sonication for 30 min. Aqueous CoCl_2_-solution (0.325%, w/v) was added drop-wisely under continuous stirring. The mixture was then sonicated for 5 min and centrifuged for another 20 min. The sediment was washed with 3% NaCl-solution and centrifuged thrice with bi-distillated water. The precipitate was left to dry at room temperature overnight, and then 10 mg was dispersed in 1 mL of THF, and the composite was characterized by SEM.

### Size distribution measurements of GCC

The size distribution of GCC was measured using dynamic light scattering (DLS). First, 1.5 mg of GCC was dispersed in 20 mL of methanol and sonicated for 30 min to create a stable suspension. Then, 1 mL of this stock dispersion was diluted with an additional 6 mL of methanol and subjected to sonication for another 15 min before analysis by DLS.

### Preparation of ion-selective electrodes (ISEs)

The ISM components were assembled in screw-cap tubes by combining 95 mg of PVC, 10 mg of either MIP or NIP, 5 mg of the ionic exchanger (TPB, K-TCPB, K-TFMPB, or PMA), and 0.2 mL of NPOE where mixed was then dissolved in 3 mL THF.

The working GCE was prepared by polishing its surface with alumina slurry in three particle sizes (1 μm, 0.3 μm, & 0.05 μm), followed by rinsing and sonication in distilled water to remove residual particles completely.

The transducer layer was settled by applying 10 µL of GCC to the electrode surface, then left to dry. Following this, 20 µL of the ISM was drop-casted over the transducer layer, allowed to dry, and conditioned overnight in 1 × 10^− 4^ M of BUP-solution. Additionally, Control sensors were Fabricated by excluding either the polymers, GCC, or both to determine the suitable exchanger and for comparison purposes.

### Standard stock and working solutions preparations

The stock solution for each drug was prepared in 20 mM phosphate buffer pH 5.0 ± 0.1 at a concentration of 1 × 10^− 2^ M. The working solutions of descendent concentrations (1 × 10^− 3^ M to 1 × 10^− 7^ M) were prepared by sequential dilutions from stock solutions. The studied interfering ions solutions were prepared, as mentioned, to get final concentrations of 1 × 10^− 4^ M.

### Application to pharmaceutical formulation

Ten Contrave^®^ tablets were subjected to whipping with cotton wetted with methanol to remove the blue film coat, then weighted, grounded, and mixed well. An amount equivalent to 6.91 mg of BUP was transferred to a 25-mL volumetric flask and sonicated with 15 mL of the buffer for 30 min. The volume was completed to obtain a final stock sample concentration of 1 × 10^− 3^ M of BUP and 6.5 × 10^− 5^ M of NAL.

### Application to spiked human plasma

ISE methods enable the direct analysis of drugs without sample pretreatment or plasma protein precipitation. However, BUP is highly bound to plasma protein (84%)^67,68^which may affect the sensitivity to the analyzed drug. Therefore, the drug was extracted, and the proteins were precipitated with methanol. An aliquot of 1 mL human plasma was spiked with 2.5 mL of 1 × 10^− 3^ M of BUP-solution and sonicated for 20 min. The plasma protein precipitation and drug extraction process proceeded with 4 mL methanol followed by sonication for another 20 min and centrifugation at 4000 rpm for 20 min. The supernatant was quantitatively transferred to a 25-mL volumetric flask and left in a fume hood for 1 h to enable the evaporation of methanol, the volume was completed with phosphate buffer pH 5 ± 0.1.

### Electrochemical impedance spectroscopy measurements (EIS)

EIS analysis was performed using a conventional three-electrode system, employing a GCE as a working electrode, Ag/AgCl reference electrode, and Pt-counter electrode. The measurements were accomplished in a solution of 0.1 M KCl and 0.01 M K_4_[Fe(CN)_6_] & K_3_[Fe(CN)_6_]. The frequency range spanned from 100 kHz to 0.1 Hz, with an alternating voltage amplitude of 5 mV. The redox behavior of the sensors was studied by cyclic voltammetry in the same solution in a potential window of -0.4 to 0.8 V.

## Results and discussion

### Preparation and characterization of the MIP

Electrochemical methods are recognized for their simplicity, speed, and minimal sample consumption generating almost no waste^19,20,69^. ISEs offer the advantage of requiring no sample preparation, allowing for a more efficient process while preserving the integrity of the sample^19,20^. Despite their selectivity, ISEs are not completely specific, especially when interfering ions have the same charge and similar characteristics^70^. In this work, the co-formulated medications, BUP and NAL, both contain basic nitrogen atoms that have pK_a_ values close to each other, BUP (8.35) and NAL (8.38), as well as comparable lipophilicity with Log P values of 3.6 and 1.92, correspondingly^67,68^. As a result, they behave as monovalent cations in acidic to slightly basic conditions, leading to significant interference that complicates their simultaneous potentiometric analysis. Although MIPs are generally produced to offer high selectivity for target analytes, they have some limitations. Imperfections in the imprinting process can lead to formation of non-specific binding sites, allowing some interference, particularly from analytes that share close sizes, shapes, or functional groups with the template^36,65,71^. Additionally, in the case of Contrave^®^ tablets, BUP is present at a much higher concentration than NAL per tablet. Also, BUP lipophilicity exceeds that of NAL based on their Log P values, so BUP is likely to cause substantial interference with NAL analysis, even when using MIP. Therefore, BUP was chosen as the target analyte to be analyzed in the presence of NAL using the MIP strategy.

To integrate BUP-MIP into the ISM, a high-yield polymerization technique, such as precipitation-polymerization, is preferred. This method generates imprinted beads with uniform size and high surface area^37^. MAA was chosen as the functional monomer due to its strong, selective interaction with templates and its acidic nature, which complements the basic amine group in BUP forming resilient hydrogen-bonds enhancing the imprinting process. UV-spectroscopy was applied to study the interaction between BUP and MAA. The spectrum of their mixture showed a marked hyperchromic shift and a marked deviation from their calculated-sum one [Fig. S2]. That confirms interaction and supports hydrogen bonds formation between the template and the monomer enhancing the drug recognition. EGDMA was selected as the cross-linker for its ability to form rigid porous polymers. A high cross-linker ratio is essential to stabilize the recognition sites; therefore a template: monomer: cross-linker ratio of 1: 4: 16 was used^40,71^. This ratio was chosen in accordance with previously established and successful protocols^40,72–74^. The experimental charachterizations including binding capacity and imprinting factor, in addition to practical measurements and selectivity studies demonstrated the practical effectiveness of this composition.

#### Scanning electron microscope (SEM) examination

**Fig. 1:**
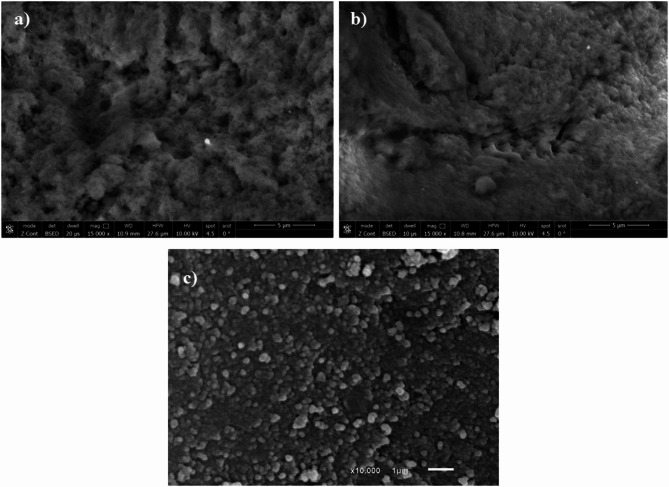
SEM surface images of: (**a**) BUP-MIP, (**b**) NIP, (**c**) GCC.

SEM imaging to examine the surfaces of the leached MIP and the NIP [Fig. 1a, b]. The images revealed that the MIP surface was rougher and more porous than the NIP surface. This spongy porous nature is associated with the imprinted holes of the leached BUP template^37,42^.

#### Brunauer-Emmett-Teller (BET) surface area and porosity analysis

The surface area and the porosity of the produced MIP and its NIP were evaluated via BET analysis. The adsorption/desorption isotherms for both MIP and NIP [Fig. S3] exhibit type IV isotherm with hysteresis loops characterizing mesoporous polymers^75^.The greater nitrogen uptake in the MIP indicates increased pore volume and surface area, consistent with the formation of imprinted sites. The specific surface area of the polymers was estimated through the isotherms’ measurements by applying the BET-equation^76^. The adsorption isotherms were further applied to determine the average pore volumes and diameters through the non-local density functional theory^77^. The results revealed that the MIP exhibits the surface area (296.22 m^2^/g) and the average pore volume (0.45 cm^3^/g) and diameter (6.06 nm) larger than those of the NIP (200.95 m^2^/g, 0.22 cm^3^/g, and 4.41 nm, respectively). These enhancements confirm the successful creation of specific binding sites through the imprinting process [Table S1].

#### Binding capacity

The binding capacity (Q) for the polymers was calculated using the following Eq. 7^8^:

$$\:\varvec{Q}=\frac{\left({\varvec{C}}_{\varvec{i}}-{\varvec{C}}_{\varvec{f}}\right)\times\:\varvec{V}}{{\varvec{M}}_{\varvec{p}\varvec{o}\varvec{l}\varvec{y}\varvec{m}\varvec{e}\varvec{r}}}$$

Where *C*_*i*_ and *C*_*f*_ are the initial and final remaining drug concentrations in mM, *V* is the prepared solution volume in L, and *M*_*polymer*_ is the added mass of the polymer in g.

The binding capacities were used to calculate the imprinting factor (IF) of the MIP by dividing its Q value by that of the NIP. The IF value (2.47) is higher than 1, which confirms the prevalence of the specific recognition in the MIP over the non-specific one in the NIP [Table S1].

Furthermore, the selectivity of the MIP was evaluated by calculating the Q values in NAL solution rather than BUP in the same concentration, where 5 mg of MIP was stirred and incubated in 0.027 mM solution of NAL in phosphate buffer pH 5 and treated as mentioned earlier. The Q-value of the selectivity evaluation ensured the binding selectivity of the MIP towards BUP [Table S1].

#### Fourier-transform infrared (FT-IR)

**Fig. 2:**
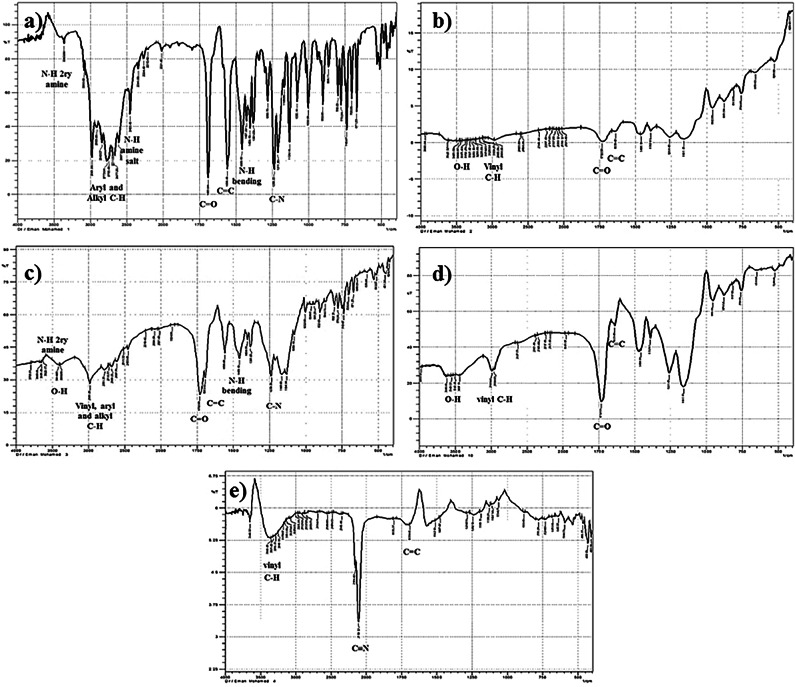
FT-IR spectra of (**a**) BUP, (**b**) leached MIP, (**c**) un-leached MIP, (**d**) NIP, (**e**) GCC.

The functional groups of the drug leached and un-leached MIPs, and the NIP were characterized with FT-IR [Fig. 2a-d]. The IR-spectrum of BUP displays a small band at 3333 cm^− 1^ corresponding to secondary amine N-H stretching vibrations along with bands at 2611 cm^− 1^ to 2522 cm^− 1^ of amine HCl salt formation. The presence of the amine group is also expressed by C-N-stretching and N-H bending vibrations at 1238 cm^− 1^ and 1458 cm^− 1^ regions, respectively. Aryl and alkyl C-H stretching appears from 2981 cm^− 1^ to 2607 cm^− 1^ also conjugated ketone C = O and vinyl C = C stretching bands are presented at 1689 cm^− 1^ and 1558 cm^− 1^, correspondingly. The spectrum of the un-leached MIP shows the representative functional groups of BUP. However, the N-H and amine salt stretching bands were hindered by the broad carboxylic acid O-H band of MAA, suggesting hydrogen bonds formation between polymerized acidic MAA and basic BUP functional groups. The polymer materials are expressed through the appearance of vinyl C-H of MAA and EGDMA with C-H bands of the drug at 2981 cm^− 1^ to 2607 cm^− 1^, which is also involved with the OH broadband. Also, the C = O and C = C bands overlapped with the presence of the same functional groups in MAA and EGDMA. The leached MIP and the NIP exhibit the same functional groups related to the polymer material. However, the bands in the NIP spectrum have higher intensity, particularly C = O band, probably due to the lack of imprinting effect in the NIP structure. All the IR spectra assured the efficacy of the polymerization, imprinting, and template leaching procedures.

### Characterization of GCC

#### SEM surface examination

SEM image [Fig. 1c] displays a rough homogenous surface with spherical particles of nanoscale size (~ 200 nm) [Fig. S4a], where the cobalt hexacyanoferrate nanoparticles cover and intercalate with the surface of graphene nano-platelets.

The morphology of the GCC composite in the SEM image, obtained without the need for conductive gold coating, may indicate the inherently good conductivity of the material. This observation agrees with the enhanced electrochemical performance demonstrated by CV and EIS measurements discussed later, supporting the role of GCC in efficient electron transfer.

#### FT-IR analysis

GCC structure was also assessed with FT-IR spectroscopy [Fig. 2e]. The spectrum demonstrates a characteristic sharp band of C ≡ N stretching at 2101 cm^− 1^, corresponding to the hexacyanoferrate group of GCC nanoparticles. It also shows a band related to GR nano-platelets carrying cobalt complex nanoparticles. Vinyl C-H and C = C stretching of GR appear as weak broad bands at 3440 cm^− 1^ to 3200 cm^− 1^ and 1560 cm^− 1^ to 1501 cm^− 1^.

### Size distribution measurements of GCC

The DLS analysis measured a zeta-average dynamic diameter of 3737 nm with a major peak at 1033 nm and a polydispersity index (PDI) of 0.598 [Fig. S4b]. That reflects the presence of large nanoplatelet-based composite, where the cobalt hexacyanoferrate nanoparticles are deposited on the graphene nano-platelets, as confirmed by SEM.

### Selection of optimum ionic exchanger

**Fig. 3:**
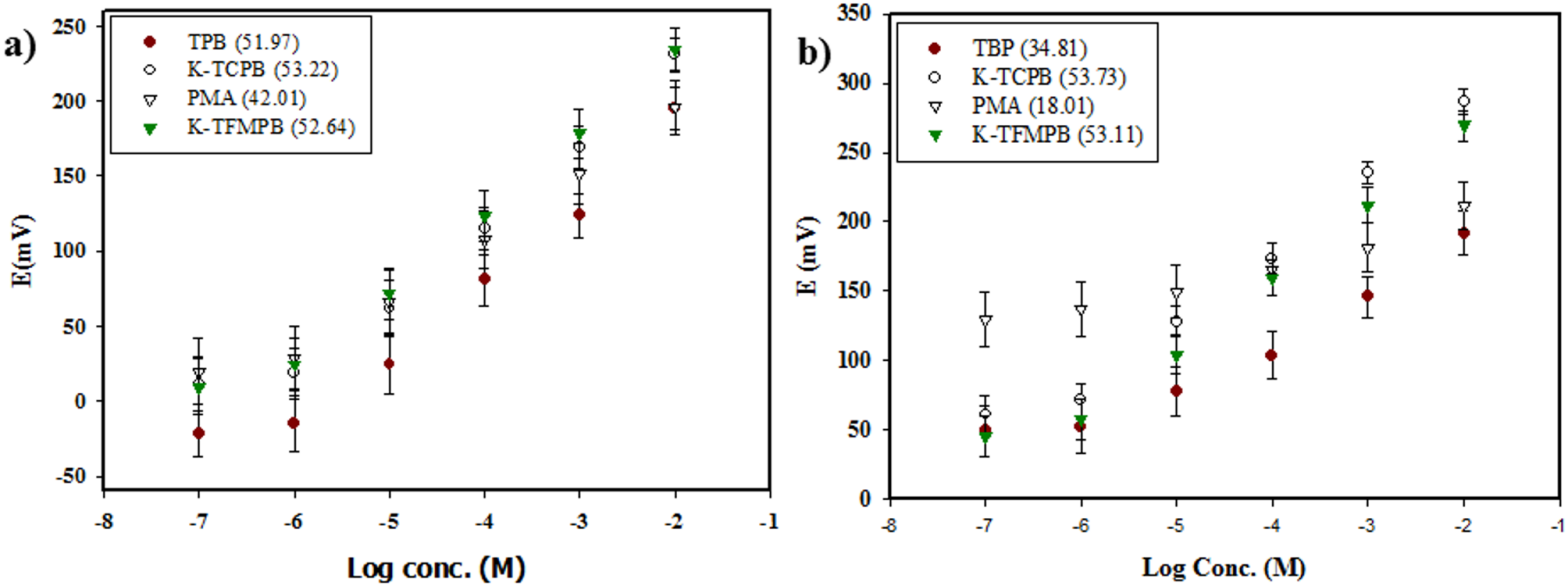
Selection of cationic exchangers (**a**) BUP, (**b**) NAL, linearity ranges (1 × 10^− 6^ to 1 × 10^− 2^ M).

In acidic to relatively neutral media, BUP is protonated at the nitrogen atom with one positive charge serving as a cation. On the surface of a bare GCE, the performance of four cationic exchangers (TPB, K-TCPB, K-TFMPB, and PMA) was evaluated for their ability to improve the response and selectivity towards BUP. Optimum slopes were obtained by using TPB, K-TCPB, and K-TFMPB. Mainly, K-TFMPB demonstrated slightly higher potential readings likely due to the combination effect of electronegativity, lipophilicity, and steric hindrance, these potential values may help to reduce interference from NAL [Fig. 3a]. Further studying of the exchangers’ performance in NAL analysis revealed that the slope obtained with TPB membrane was relatively low compared with the more lipophilic exchangers [Fig. 3b]. This finding suggests a poor response of TPB to increasing concentrations of NAL. Also, these results support further studies with K-TFMPB due to its high response to BUP and with TPB because of its inadequate response to NAL. The observed differences in responses between the examined drugs and cationic exchangers can be attributed to the individual interaction between the ions of each drug and the exchangers. These interactions depend on the mutual compatibility of their molecular structures, which involve factors such as steric hindrance, interaction with other functional groups in the drug’s structure, and shared electronegativity and lipophilicity.

### Performance of the studied sensors

**Fig. 4:**
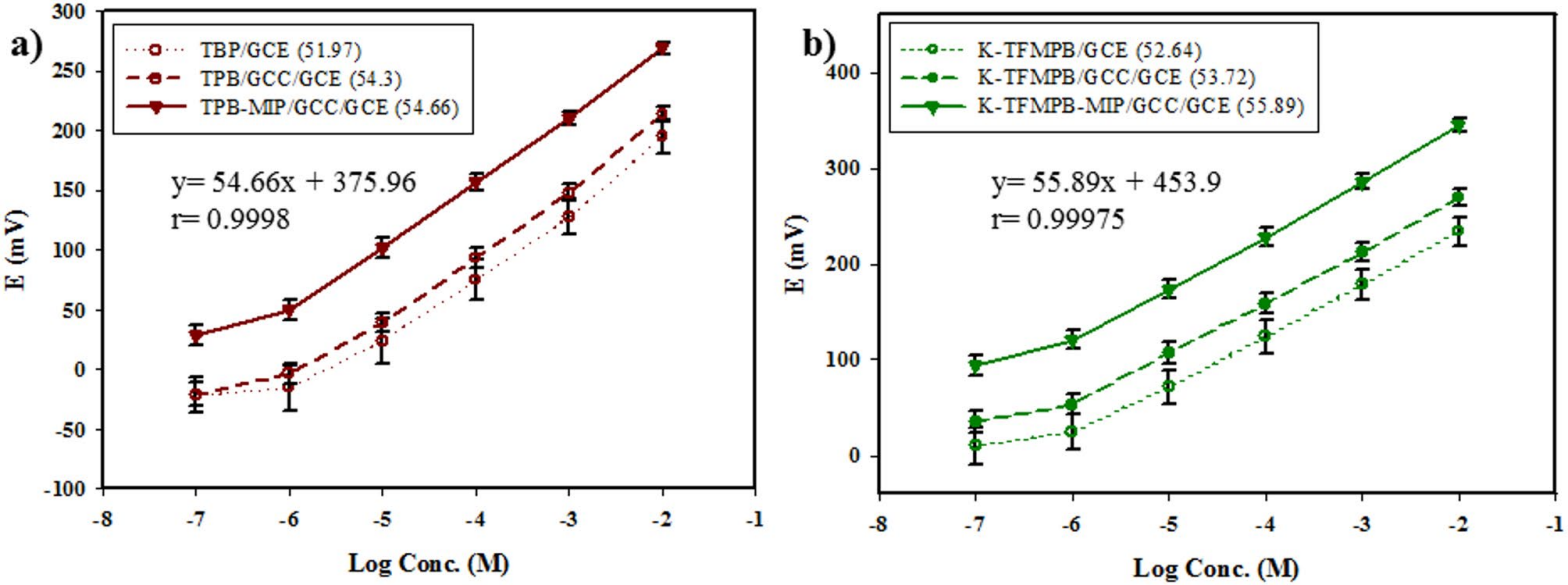
Potential profile in mV vs. log concentration of BUP by: (**a**) TPB sensors, (**b**) K-TFMPB sensors, with their corresponding slopes and equations of MIP-modified sensors.

The detection mechanism of the proposed potentiometric sensors is based on phase boundary potential. In this mechanism, BUP ions partition into the selective membrane, altering the boundary potential at the membrane/aqueous interface. The observed potential change is related to the ion activity (in logarithmic function), consistent with Nernstian behavior of monovalent cations^79^. The electrochemical performances of the proposed sensors towards BUP are summarized in Table 1 according to the IUPAC-recommendations^80,81^and the profiles of the potentials along with the straight line equations of the MIP-modified sensors are presented in Fig. 4. A GCC layer introduced as a hydrophobic transducer between the ISMs and GCE. The unmodified sensors produced less precise results (%RSD more than 2) and shorter stability work period likely due to ion flux into the aqueous layer. GCC-modified sensors displayed better Nernastian response and precision, lower LOD, response time, and potential drift than GCC-free sensors. Repeatability was verified *via* intraday measurements with three different concentrations measured in triplicates, the resulted %RSDs are less than 2. The stability of the proposed sensors was tested over a time period (up to 50 days). The GCC-modified sensors retained over 90% of its initial potential responses (Table 1). That can be attributed to the transducer layer of GCC nanoparticles, which combines the hydrophobicity and conductivity of graphene and the electroactivity and redox properties of cobalt hexacyanoferrate nanoparticles^27^. Subsequently, BUP-MIP was incorporated into the composition of the ISMs to enhance selectivity. The effect of varying the mass of the MIP on the sensors’ performance and selectivity was evaluated. Different amounts of BUP-MIP, specifically 1 mg, 2 mg, 5 mg, 10 mg, 15 mg, and 20 mg, were separately added to the components of TPB-ISM and K-TFMPB-ISM. Gradually increasing the MIP amount up to 10 mg led to improvements in the sensors’ overall performance such as slope, selectivity, and precision. However, no significant enhancements were observed with 15 mg. At 20 mg, non-uniform MIP particles dispersion and a denser ISM surface were observed. These caused a reduction in the slopes and response potential of the sensors. As a result, 10 mg of the MIP was selected for the rest of the study. The observed improvements in performance and selectivity can result from the specific binding sites within the synthesized MIP that improved drug recognition.

**Table 1:** Electrochemical responses of the proposed MIP/GCC-modified sensors compared with MIP free-GCC-modified and unmodified sensors.

Parameter	TPB/GCE			K-TFMPB/GCE		
	MIP/GCC	GCC	Plain GCE	MIP/GCC	GCC	Plain GCE
Slope (mV/decade)^a^	54.66	54.30	51.97	55.89	53.72	52.64
Intercept (mV)	375.96	315.36	289.6	453.9	374.92	337.04
Correlation coefficient (r)	0.9998	0.9973	0.9949	0.99975	0.9998	0.99945
Concentration range (M)	10^− 6^ to 10^− 2^	10^− 6^ to 10^− 2^	10^− 6^ to 10^− 2^	10^− 6^ to 10^− 2^	10^− 6^ to 10^− 2^	10^− 6^ to 10^− 2^
LOD (M)^b^	2.51 × 10^− 7^	5.01 × 10^− 7^	8.91 × 10^− 7^	2 × 10^− 7^	3.98 × 10^− 7^	7.94 × 10^− 7^
Stability (Days)	45	45	35	50	50	40
Response time(s)	5–10	5–10	15–20	5–10	5–10	15–20
Working pH range	3–6	3–6	3–6	3–6	3–6	3–6
Accuracy (Mean ± SD)^c^	100.64 ± 1.38	100.39 ± 1.43	100.10 ± 2.07	98.44 ± 1.35	99.56 ± 1.54	100.20 ± 2.39
Precision						
(RSD%)^d^	0.95	1.22	1.83	1.22	1.39	1.95
(RSD%)^e^	1.10	1.48	2.14	1.53	1.63	2.23
Reproducibility (RSD%)^f^	1.91	–	–	1.86	–	–
Dosage form (Recovery% ± SD)^a^	100.83 ± 0.86	–	–	99.94 ± 0.93	–	–
Plasma (Recovery% ± SD) ^a^	96.81 ± 1.62	–	–	95.78 ± 1.18	–	–

^a^Average of three determinations.

^b^Limit of detection (measured by interception of the extrapolated arms of potential profiles figures.

^c^Mean ± SD of three concentrations measured in triplicates.

^d^Intraday precision or repeatability (average of three different concentrations measured in triplicates in the same day).

^e^Interday precision or intermediate precision (average of three different concentrations measured in triplicates for three days).

^f^Average of three different concentration with three batches of the sensors.

The response time is the interval between the moment the proposed sensors and the reference electrode come into contact with the sample solution and the point at a stable signal (± 1 mV) is achieved. For the unmodified sensors, the response time ranged from 15 s to 20 s. In contrast, the GCC-modified and MIP/GCC-modified sensors exhibited a shorter response time of 5 s to 10 s. This rapid response can be attributed to the absence of a water layer, which prevents equilibrium delays.

### Influence of GCC transducer layer on GCE

The inclusion of the GCC-layer as a transducer enhanced and stabilized the potential response in the modified sensors and reduced the potential drift observed over time. This improvement is due to the GCC-layer’s role in blocking the accumulation of a aqueous layer between the GCE-surface and the ISM, as well as facilitating better charge transfer across the sensing layers. The enhanced performance of GCC-layers could be attributed to the synergistic interaction between GR and CoNP. GR provides a highly conductive and hydrophobic platform that facilitates charge transfer and inhibits aqueous interlayer formation. Meanwhile, CoNP provides redox-active sites that enhance ion-to-electron conversion and stabilize the interfacial potential. The combination of both materials within the GCC structure offers complementary advantages; enhanced conductivity which improves signal transduction and expanded hydrophobicity that limits the moisture-induced potential drift, resulting in a more stable and reproducible electrode response. This cooperative effect highlights the added value of using dual-component composite rather than individual materials.

#### Aqueous layer test

**Fig. 5:**
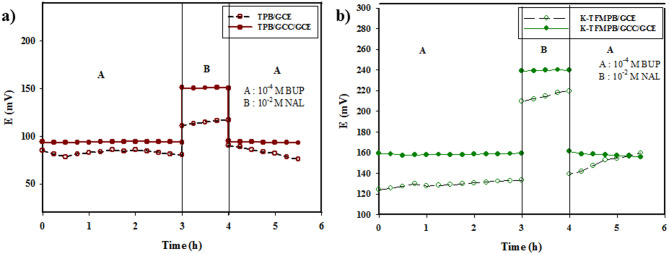
Potential drift and Water layer test for: (**a**) TPB sensors, (**b**) K-TFMPB sensors, using GCC-modified and unmodified sensors.

The aqueous layer test is used for long-term potential stability determination. It is based on measuring the potential drift after changing from 1 × 10^− 4^ M BUP solution to a higher concentration of interfering ion (NAL) solution, 1 × 10^− 2^ M, then back to BUP solution. The presence of an aqueous layer between the ISM and the underlying electrode may cause a potential drift due to changes in its composition by ion flux through the membrane. The unmodified GCEs exhibited a significant potential drift even at the first three hours in BUP solution, while the GCC-modified GCEs showed a more stable potential over time [Fig. 5]. This ensures the absence of an aqueous layer under the ISM due to graphene hydrophobicity and the ions-to-electrons conductance of the formed nanoparticles.

#### Impedance study and redox behavior

The GCC transducer layer was characterized by EIS. Bode plots for both unmodified and GCC-modified sensors demonstrated a decrease in transfer resistance in the GCC-modified sensors, which improved charge transfer efficiency [Fig. S5 a, b]. Cyclic voltammograms (CV) of both unmodified and GCC-modified sensors revealed a significant enhancement in the current response of the modified sensors compared to the unmodified ones. This improvement could be attributed to enhanced conductivity and more stable charge transfer at the sensors’ interface [Fig. S5 c, d].

### Assessing sensors’ performance at various pH values

To achieve a sensitive quantitative assay, a pH study covering pH ranges of 2 to 11 on 1 × 10^− 4^ M and 1 × 10^− 3^ M BUP solutions was done to explore the most suitable measurement conditions. The MIP responses were almost steady at a pH range of 3 to 6, with a change between measured concentrations close to the developed slope mV. There was a gradual decrease in the measured potential with increasing pH without a constant region [Fig. S6]. Therefore, a buffer solution with a pH value of 5 was adopted in the analysis of BUP by the proposed sensors to ensure the best stable and reproducible response.

### Sensors’ selectivity

**Fig. 6:**
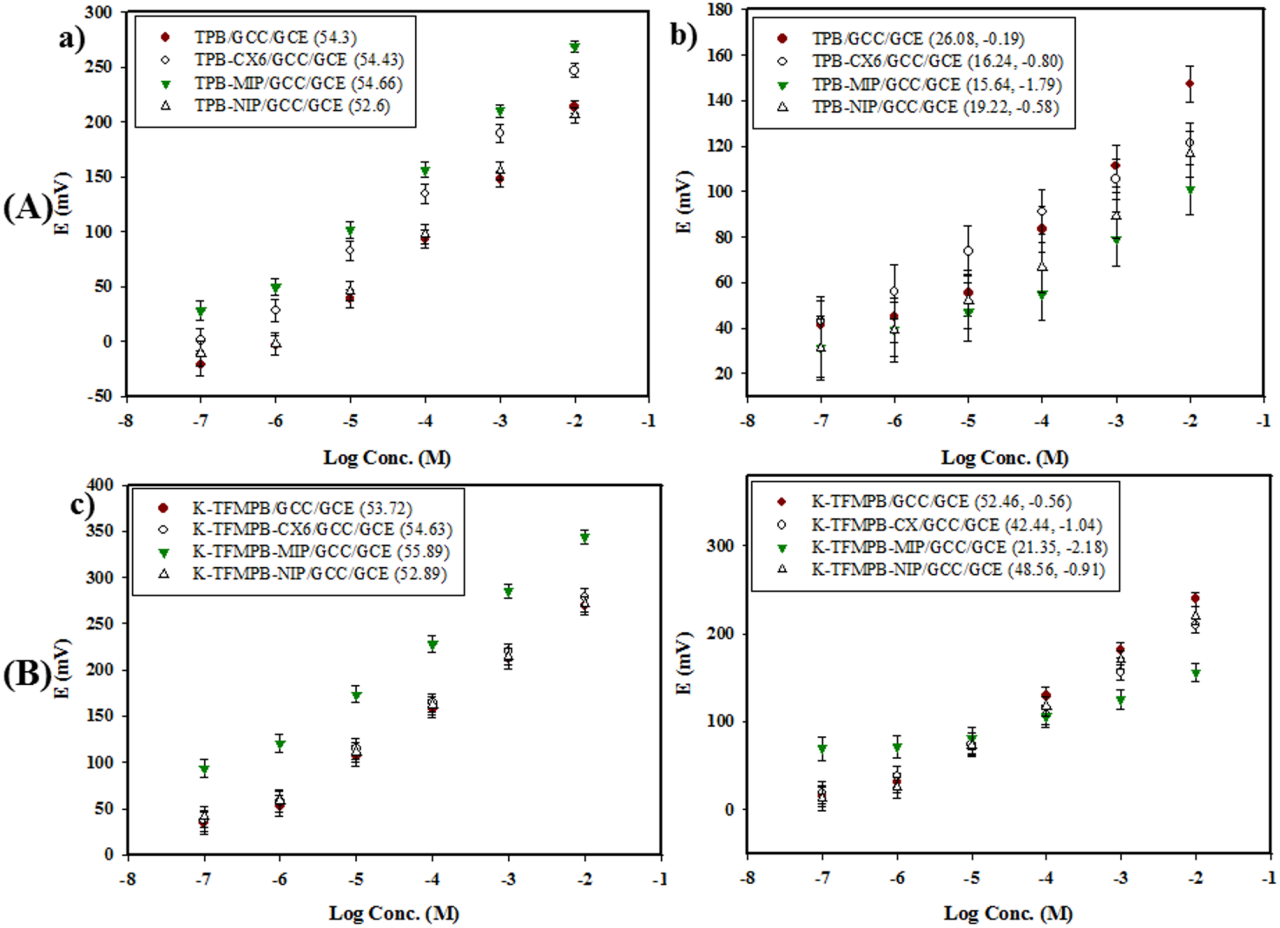
Response of various sensors of: (**A**) BUP-TPB as function of log concentration of (a) BUP and (b) NAL, (**B**) BUP-TFMPB as function of log concentration of (c) BUP and (d) NAL, where values between parentheses represent the slope in (a, c) figures, and slope and (Log K^pot^_Drug, I_) in (b, d) figures.

The specificity and selectivity of the proposed sensors were examined through separate solution method (SSM) by measuring the potential of 1 × 10^− 4^ M BUP and the equal concentration of interfering ions (NAL, variety of cations, as well as some commonly used additives). The unbiased potentiometric selectivity coefficient (K^pot^_primary ion, interferent_) was used to assess the selectivity of the proposed MIP/GCC-modified sensors [Table 2], calculated by the equation: log (K^pot^_primary ion, interferent_) = - (E_1_-E_2_) / S, where *E*_*1*_ is the measured potential of 1 × 10^− 4^ M of BUP, *E*_2_ is the measured potential of the interfering ion at the equal concentration and *S* is the slope of the sensor. Firstly, MIP/GCC-modified sensors were compared with the matching GCC-modified sensors, NIP/GCC-modified sensors, and CX6/GCC-modified sensors (an example of an ionophore-decorated sensor)^82,83^. This comparison was based on different sensors’ performance with BUP [Fig. 6a, c] and selectivity towards NAL [Fig. 6b, d] using calibration curves to determine the slope and selectivity coefficient in each case.

**Table 2:** Selectivity coefficient (Log K^pot^_Drug, I_) of the proposed sensors using separate solution method.

Interferent (I)	Log K^pot^_BUP, I_	
	TPB-MIP/GCC/GCE	K-TFMPB-MIP/GCC/GCE
NAL	− 1.79	− 2.18
Na^+^	− 2.80	− 3.35
K^+^	− 2.93	− 3.33
Ca^2+^	− 3.01	− 3.42
Mg^2+^	− 3.06	− 3.40
Glucose	− 3.11	− 3.56
Lactose	− 3.15	− 3.60
Starch	− 3.23	− 3.64

While the incorporation of CX6 as an ionophore enhanced sensors’ performance and selectivity, the MIP-modified sensors exhibited noticeably improved slopes, lesser detection limits, and higher selectivity (by approximately two orders of magnitude) compared to the other sensors. This confirms the superiority of the MIP for the analysis of BUP in the mixture. The findings showed that the MIP increased the selectivity for BUP over NAL and considerably reduced the sensors’ response to NAL, as reflected in the lower slopes and the nearly flat calibration curves. It suggests that a specific binding to BUP prevails over non-specific interactions in the MIP. It is important to note that BUP is considered the main component according to the ratio of both drugs in the studied tablet. Thus, the minor interference from NAL which was indicated by the slightly lower selectivity coefficient in TPB-MIP/GCC/GCE sensor, was unlikely to affect the accuracy of BUP analysis. Additionally, the selectivity of the MIP/GCC-modified sensors against some inorganic cations and commonly used additives was evaluated [Fig. S7]. The selectivity coefficients revealed no significant interference from these substances, probably due to differences in the ionic size and lipophilicity of the cations and the absence of charge in the additives.

### Applications to Contrave^®^ tablets and human plasma

The studied MIP/GCC-modified sensors were used to determine BUP in its tablet formulation Contrave^®^ and extended to human plasma to evaluate the sensors’ efficiency in real-life samples. The results obtained in Table 1 show that there is no interference from the co-formulated drug (NAL) or the excipients present in the formulation. Additionally, the sensors successfully determined BUP in the spiked human plasma extraction. The sensors can be used to assay BUP in its pharmaceutical tablets without prior treatment steps besides in human plasma after protein precipitation and extraction with methanol due to BUP’s high binding to plasma proteins.

### Statistical evaluation of the proposed method

So as to evaluate the reliability of the proposed sensors, their results were compared to a reported method^51^. The calculated student’s t-test and F-test values displayed no significant difference between the proposed MIP/GCC-modified sensors and the reported method [Table S2]. Moreover, the proposed sensors demonstrated lower LOD values of 2.51 × 10^− 7^ M for TPB-MIP/GCC/GCE and 2 × 10^− 7^ M for K-TFMPB-MIP/GCC/GCE compared with 8.5 × 10^− 6^ M for PVC-membrane and 3.5 × 10^− 6^ M for carbon paste electrode presented in the reported potentiometric method^57^.

### Green profile and whiteness assessment metrics

The Increasing focus on environmentally friendly methods has gained considerable attention in the analytical chemistry field^19,54,84–87^. To weigh the environmental and sustainability aspects of the proposed method, tools such as AGREE, WAC, and MoGAPI were employed [Table 3].

**Table 3:**
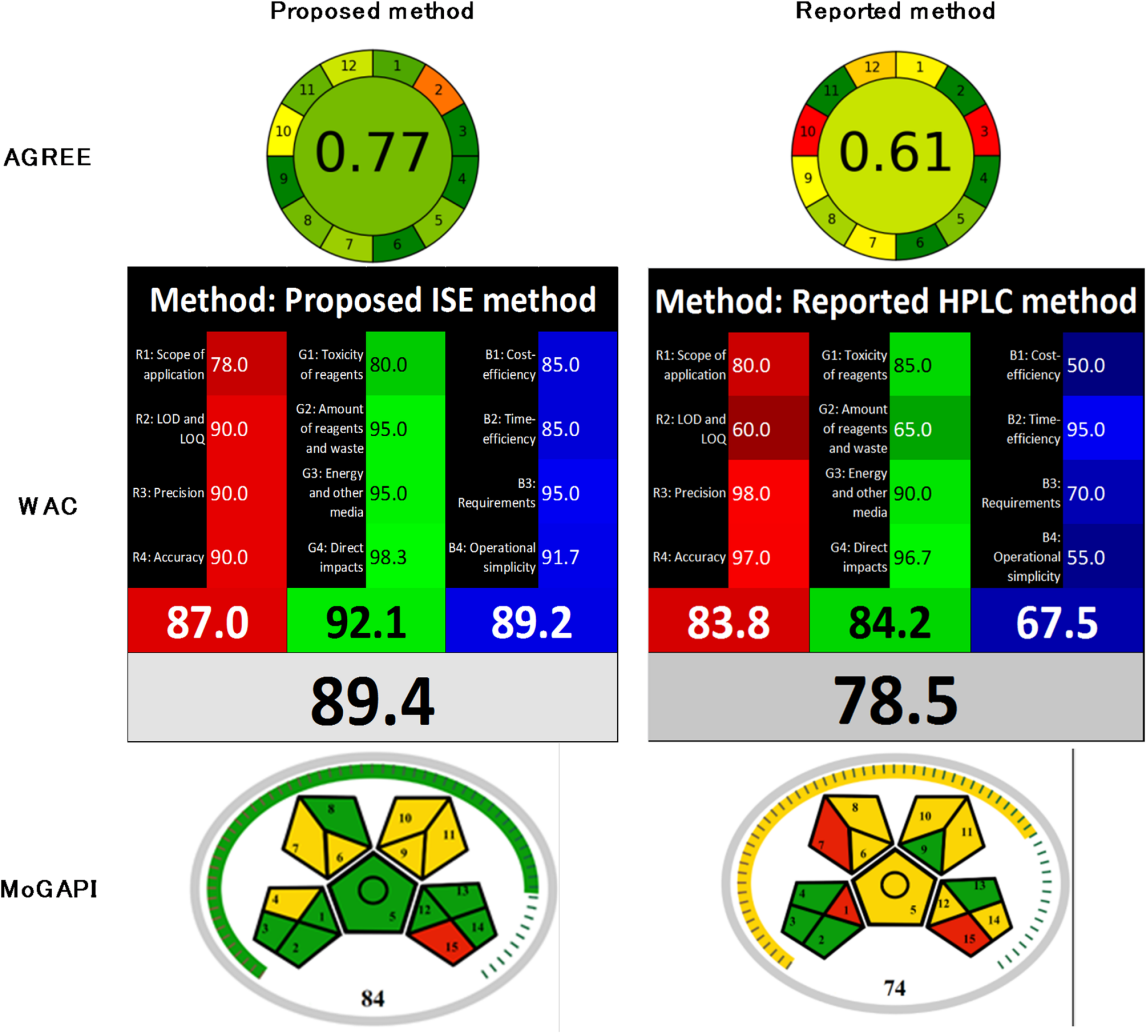
Greenness and whiteness assessment comparison between the proposed and reported methods.

The AGREE tool offers a clock-like pictogram that quantifies and visually represents the method’s compliance with the 12 principles of green analytical chemistry. It assigns a score between 0 and 1, with higher scores reflecting greater alignment with the green principles^62^. The proposed method scored 0.77, in contrast to 0.61 for the reported method.

The WAC assesses the overall sustainability of the method through a Red/Green/Blue (RGB) model. It evaluates three key aspects: analytical performance (red), environmental and safety impacts (green), and operational and economic viability (blue). These metrics are combined to produce a color shade from black to white and a numerical score ranging from 0 to 100. A higher score and a whiter shade indicate greater sustainability^63^. The proposed method earned a score of 89.4, outperforming the reported method’s score of 78.5.

MoGAPI offers a segmented pictogram with coded sections red, yellow, and green representing low, medium, and high environmental safety, correspondingly. This tool is supplied with a free online software that calculates a total score for more accessible methods’ comparison and labels each segment for clearer follow-up^64^. For the proposed method, the assessment mostly displayed green segments, reflecting a low ecological impact, with some yellow segments and one red segment for the absence of waste management. The generated total scores of the proposed and reported methods are 84 and 74, respectively, supporting the proposed method’s greener profile than the reported one.

In conclusion, the proposed method demonstrated superior environmental and sustainability metrics than the reported one, with higher scores in both greenness and whiteness evaluations.

## Conclusion

This work involves MIP-customized sensors for the selective potentiometric assay of BUP in the presence of NAL in their co-formulated tablet, controlling food cravings, and in spiked human plasma. The selective sensing capability is governed by the imprinted cavities within the MIP, which from synthetic receptors for the target drug, BUP, enabling specific binding and recognition. To evaluate this virtue, rebinding studies using FT-IR analysis were conducted and compared with leached/template-free MIP, alongside measurements of rebinding capacity. The potentiometric analysis further revealed substantial enhancement in the performance of the MIP-modified sensors, particularly in terms of selectivity, slopes, and detection limits towards NAL compared to MIP-free sensors. The MIP demonstrated a remarkable selectivity effect, improving the detection limits and selectivity for BUP while effectively suppressing the response to NAL. Additionally, the sensors were integrated with GCC as a transducer layer. Implementing GCC ensured reproducible, stable, and rapid sensor responses by preventing the formation of an aqueous layer underneath the ISM. The study proposes the use of the proposed method as an equipment-less, fast, intuitive, and sustainable alternative for selective analysis of BUP.

## Supplementary Information

Below is the link to the electronic supplementary material.

Supplementary Material 1

## Data Availability

All data generated or analyzed during this study are included in this article.
