# Supplementary material for: Graphene-cobalt hexacyanoferrate modified sensor doped with molecularly imprinted polymer for selective potentiometric determination of bupropion
Source: Sci Rep. 2025 Aug 22;15:30892. doi: 10.1038/s41598-025-16259-z (PMC12373795; doi:10.1038/s41598-025-16259-z)
Supplement: Supplementary file 1 — Supplementary Material 1 [file 41598_2025_16259_MOESM1_ESM.docx]

Graphene-Cobalt Hexacyanoferrate Modified Sensor Doped with Molecularly Imprinted Polymer for Selective Potentiometric Determination of Bupropion

Eman M. Moaaz*, Ahmed S. Fayed, Mamdouh R. Rezk, Ezzat M. Abdel-Moety

*Pharmaceutical Analytical Chemistry Department, Faculty of Pharmacy-Cairo University, Kasr El-Aini Street, ET-11562 Cairo, Egypt*

* Corresponding author email: [eman.moaaz@pharma.cu.edu.eg](mailto:eman.moaaz@pharma.cu.edu.eg)


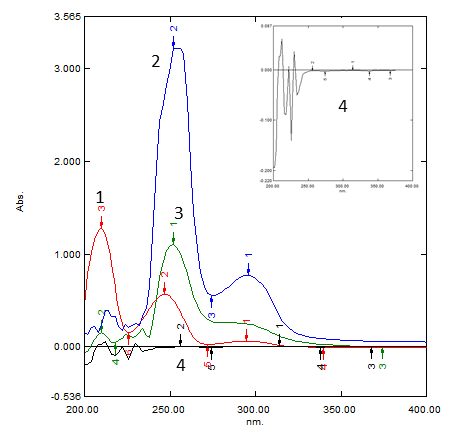


Figure S1. UV spectra of 1) BUP (0.05 mM) in methanol, 2) first wash, 3) medium wash, and 4) last wash.


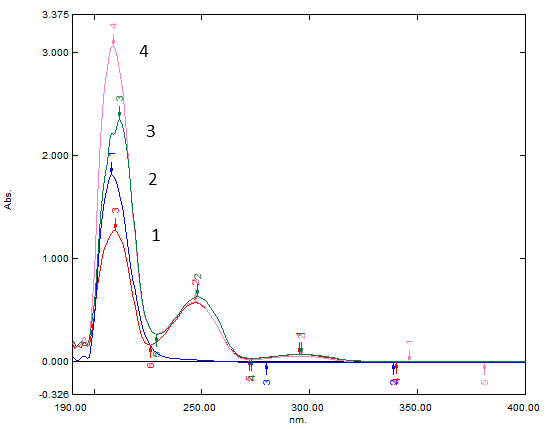


Figure S2. UV spectra in methanol of 1) BUP (0.05 mM), 2) 0.2 mM MAA, 3) actual mixture of BUP (0.05 mM) and MAA (0.2 mM), and 4) their calculated mixture.


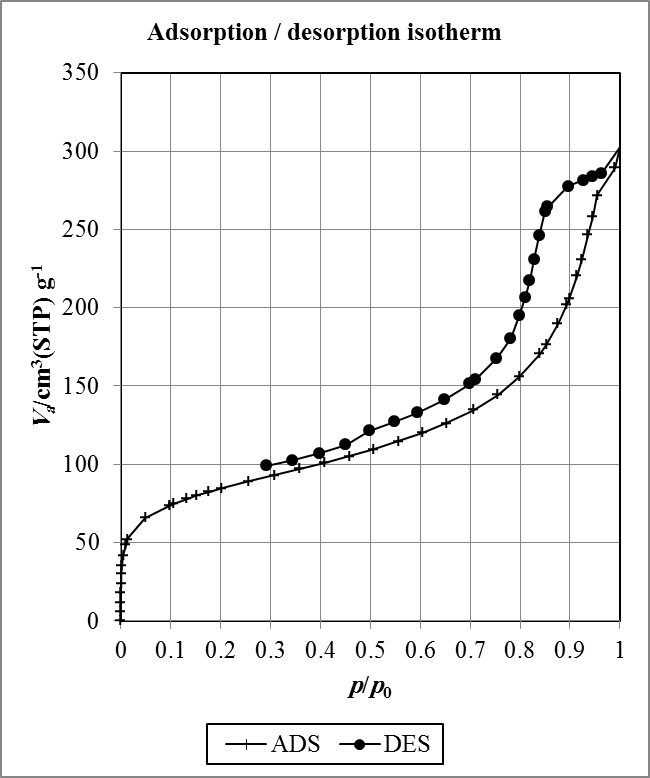

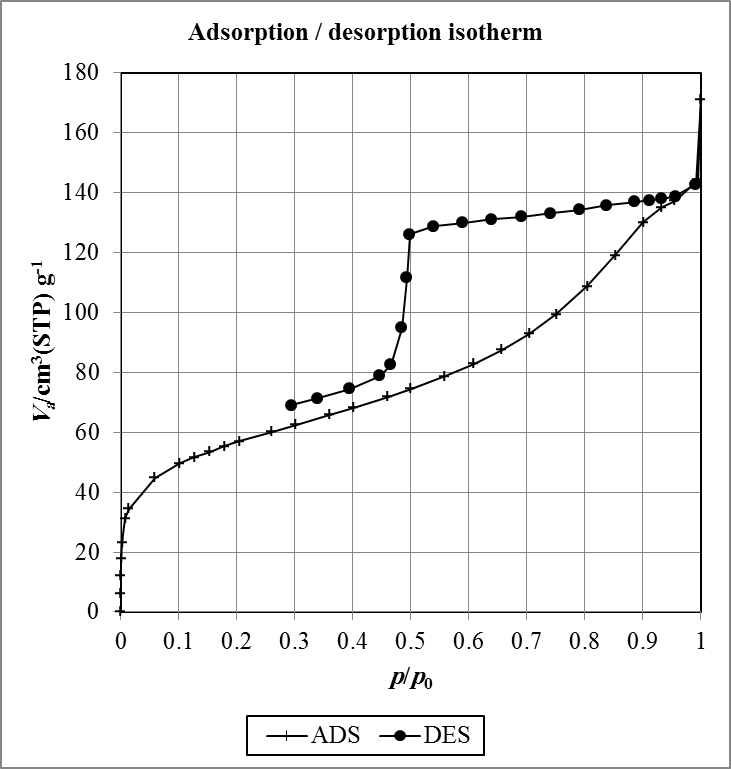


b)

a)

Figure S3. Nitrogen adsorption/desorption isotherm of a) BUP-MIP, b) NIP.


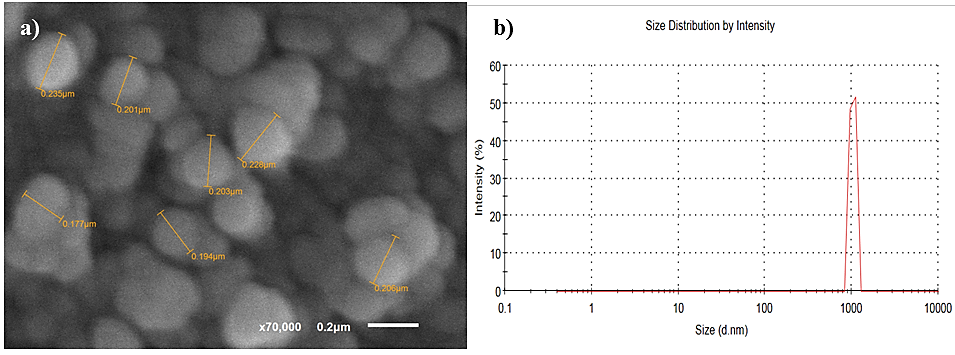


Figure S4. a) SEM measurements of the precipitated cobalt hexacyanoferrate nanoparticles on the graphene nano-platelets, b) DLS size distribution graph of GCC dispersed in methanol.


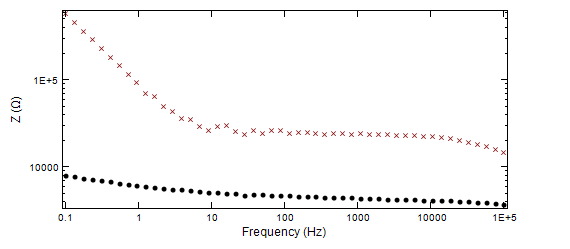

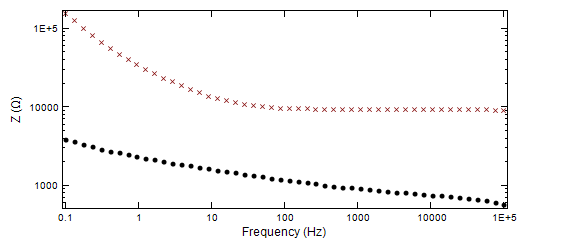


b)

a)


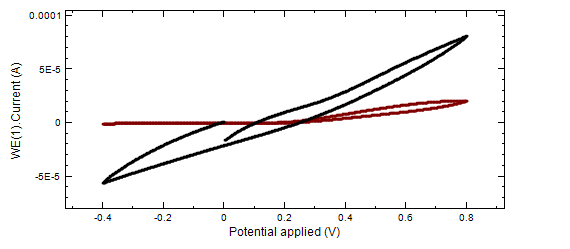

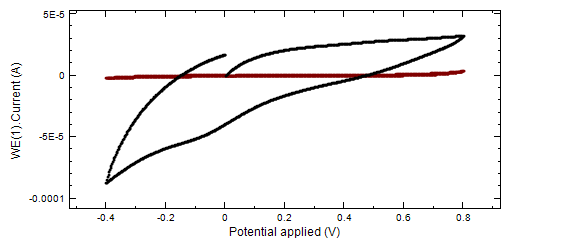


d)

c)

Figure S5. Bode plots of GCC-modified (….) and unmodified (xxxx) GCEs of: a) TPB sensors, b) K-TFMPB sensors, CV of GCC-modified (ــــــ) and unmodified (ــــــــــ) GCEs of: c) TPB sensors, d) K-TFMPB sensors.

b)

a)

Figure S6. Effect of pH on the *emf* of the proposed MIP/GCC/GCE sensors: a) TPB sensor, b) K-TFMPB sensor.


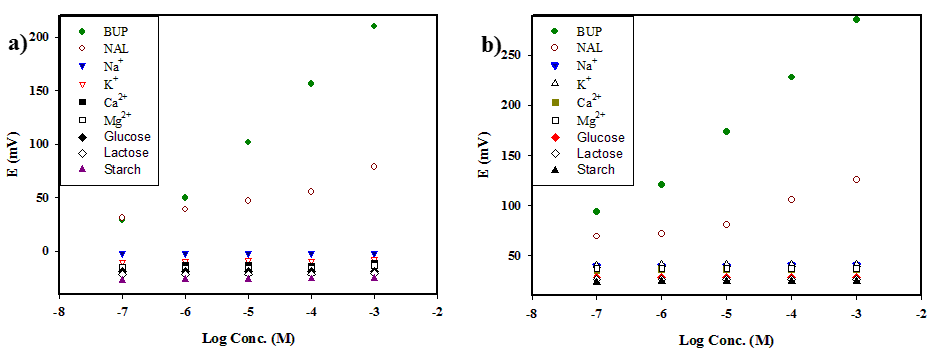


Figure S7. Calibration plots of BUP, the co-formulated drug (NAL), common inorganic cations, and common additives by: a) TPB sensor, b) K-TFMPB sensor.

Table S1. Results of BET analysis and binding capacity calculations for the MIP and the NIP.

| **Polymer** | **Specific surface area (m^2^/g)** | **Pore Volume (cm^3^/g)** | **Average Pore Diameter (nm)** | **Binding Capacity Q (mmol/g)** | **Imprinting factor** | **Selectivity Evaluation by**  **Q (mmol/g)** |
| --- | --- | --- | --- | --- | --- | --- |
| **MIP** | 296.22 | 0.45 | 6.06 | 0.0533 | 2.47 | 0.024 |
| **NIP** | 200.95 | 0.22 | 4.41 | 0.0216 | NA | - |

Table S2. Statistical comparison of the results obtained by the proposed sensors and the reported method.

| **Value** | **TPB-MIP/ GCC /GCE** | **K-TFMPB-MIP/ GCC /GCE** | **Reported method^a^** |
| --- | --- | --- | --- |
| **Mean** | 99.89 | 100.23 | 99.86 |
| **SD** | 1.20 | 1.04 | 1.63 |
| **%RSD** | 1.20 | 1.04 | 1.63 |
| **Variance** | 1.44 | 1.08 | 2.66 |
| **N** | 5 | 5 | 5 |
| **t-test^b^ (2.306)** | 0.033 | 0.428 |  |
| **F Value^b^ (6.39)** | 1.845 | 2.456 |  |

^a^ RP-HPLC method with mobile phase composed phosphate buffer (pH 3) and acetonitrile in ratio of 60: 40. The flow rate was adjusted at 1 mL/min and UV detection at 224 nm.
^b^ The values in the parenthesis are the corresponding theoretical values of t and F at P = 0.05.
